# Metal Oxides and Ion-Exchanging Surfaces as pH Sensors in Liquids: State-of-the-Art and Outlook

**DOI:** 10.3390/s90604955

**Published:** 2009-06-23

**Authors:** Peter Kurzweil

**Affiliations:** University of Applied Sciences, Kaiser-Wilhelm-Ring 23, D-92224 Amberg, Germany; E-Mail: p.kurzweil@haw-aw.de; Tel.: +49-9621-482-154; Fax: +49-9621-482-145

**Keywords:** pH sensor, platinum metal oxides, RuO_2_, IrO_2_, reference electrode, hydrogen electrode, capacitance

## Abstract

Novel applications of online pH determinations at temperatures from -35 °C to 130 °C in technical and biological media, which are all but ideal aqueous solutions, require new approaches to pH monitoring. The glass electrode, introduced nearly hundred years ago, and chemical sensors based on field effect transistors (ISFET) show specific drawbacks with respect to handling and long-time stability. Proton sensitive metal oxides seem to be a promising and alternative to the state-of-the-art measuring methods, and might overcome some problems of classical hydrogen electrodes and reference electrodes.

## 1. Introduction

Although Sørensen's concept of pH dates back to the beginning of the 20th century, the measurement of absolute pH values in aqueous and non-aqueous solutions still proves most circumstantial by means of a Harned cell. Therefore all known proton probes, such as the glass electrode and Ion Selective Field Effect Transistors (ISFETs) are based on calibration steps based on standard solutions. Endeavors for replacing the potentiometric method by coulometric proton counting still appear futuristic. The scope of this review article comprises:

(i) The analytical meaning of the pH value, (ii) traditional and novel pH measuring techniques, and (iii) the variety of pH sensitive materials and (iv) preparation methods described in the literature.
Platinum metal oxides are presented as materials which are able to replicate similar proton-exchange processes occuring at common glass membranes. This justifies the title “ion-exchanging surfaces”, because the bulk of platinum metal oxides is mainly electronically conducting. Less expensive than commercial glass electrodes, disposable metal oxide probes are useful, e.g., for applications in aqueous and biological media.
Additionally, pH dependent redox processes occur at platinum metal oxides. With respect to a future direct pH indicator, the redox pseudocapacitance of hydrous RuO_2_ is considered as a model system, which requires the passage of protons through grain boundaries and cracks on the porous electrode surface for the establishment of the equilibrium potential.
Finally, this article points out the role of (i) the support material, and (ii) the reference electrode. The pH sensitive material is usually coated on a support material to create a durable electrode.

## 2. pH Monitoring Using Glassy Materials and Similar Proton Probes

The term pH value was coined in 1909 by Søren P.L. Sørensen [1] to describe the solution pressure *p*_H_ = –lg (*c*_H_/mol dm^–3^), pondus Hydrogenii or potentia Hydrogenii, of hydrogen ions in aqueous solutions. IUPAC defines the quantity pH in terms of the molality basis activity *a*_H_ of hydrogen ions in solution:

(1) $$p\text{H}=-\mathrm{lg}a_{\text{H}}=-\mathrm{lg}\frac{m_{\text{H}}\cdot \gamma_{\text{H}}}{m^{0}}=-\mathrm{lg}\frac{c_{\text{H}}\cdot \gamma_{c\text{H}}}{c^{0}}+\mathrm{lg}\frac{\rho }{{\text{gcm}}^{-3}}$$

where γ_H_ denotes the molal activity coefficient of the hydrogen ion H^+^ at the molality *m*_H_ (in mol per kilogram of *solvent*), and *m*^0^ ≡ 1 mol kg^–1^. The IUPAC definition holds although free hydrogen ions cannot exist in aqueous solution; *a*_H_ denotes as well the activity of hydronium ions H_3_O^+^ and higher associates [H_3_O·*n*H_2_O]^+^, *n* = 1,2,3,4,5,6. For calculations with volume concentrations, the density of pure water ρ must be corrected: *p*H*_c_* = *p*H – lg ρ. As the single ion activity *a*_H_ cannot be measured by any thermodynamically valid method, primary pH standards were established.

Precise pH measurements [2] rely on the potentiometric method [3] rather than on optical methods.

### 2.1. Glass Electrodes

**Figure 1.**
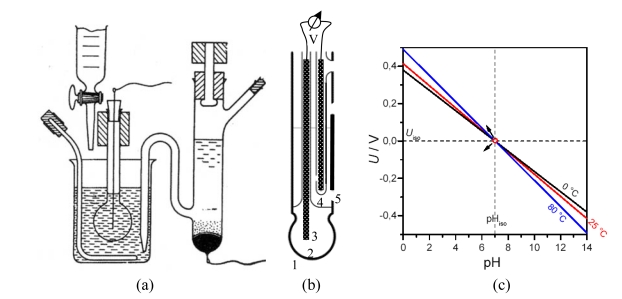
(a) Experimental setup by Haber and Klemensiewicz. (b) Single-rod measuring cell with double junction: 1 = solution, 2 = inner electrolyte (KCl, 3 mol/L, pH 7), 3 = reference electrode, 4 = external Ag|AgCl|KCl reference electrode, 5 = junction. (c) Ideal chain voltage versus pH.

In 1909, based on observations of Cremer (1906) at glass membranes, F. Haber and Z. Klemensiewicz developed the pH glass electrode [4]: a glass bubble filled with strong electrolyte and a silver|silver chloride electrode inside (Figure 1-a). Improved pH selective glasses were found by Mcinnes in 1930. Present glasses contain, e.g., 63% SiO_2_, 28% Li_2_O, 5% BaO, 2% La_2_O_3_ [5]. UO_2_ and TiO_2_ improve the performance in alkaline solutions [6]. A. Beckman's practical pH meter (1934) used a high-gain vacuum tube amplifier in order to replace the earlier used sensitive galvanoscope by a cheap milliamperemeter. Swiss chemist W. Ingold (1952) created the single-rod measuring chain, which finally combined working electrode and reference electrode in one shaft. Advanced potentiometric measuring chains have become commercially available since the 1970s. In 1986, Ingold replaced the liquid inner electrolyte by a gelled electrolyte, in order to slow down the loss of electrolyte through the junction between reference electrode and external solution, however, at the cost of lifetime.

The double junction electrode introduced an additional chamber between reference electrode and external solution to save the reference electrolyte from external contamination (Figure 1-b). The potential difference Δφ_I–II_ across the thin glass membrane reflects the difference between the H^+^ activities *a* on both sides, and can only be determined by means of two reference electrodes. The less than 0.5 μm thick soaking layers on both sides of the membrane enable the exchange of cations in the silicate framework against H_3_O^+^ from the surrounding solutions and vice versa. The two soaking layers are connected by the cationic conductivity of the thin glass membrane. The measured chain voltage *E* is made up of the membrane contribution and the diffusion potentials at the liquid junction between each reference electrode and the ambient solution:

(2a) $$\Delta \phi_{I-II}=\frac{RT}{F}\ln\frac{a{(\text{H}}^{+},\text{glass I})}{a({\text{H}}^{+},\text{solutionI})}-\frac{RT}{F}\ln\frac{a{(\text{H}}^{+},\text{glass II})}{a({\text{H}}^{+},\text{solutionII})}=\frac{RT}{F}(p{\text{H}}_{II}-p{\text{H}}_{I})+\Delta \phi_{as}$$

(2b) $$E=\frac{RT}{F}(p{\text{H}}_{\text{II}}-p{\text{H}}_{\text{I}})+E_{as}+E_{d}$$

The measured voltage *E* consists of (1) the H^+^ activity-dependent potential drop Δφ_1_ between glass membrane and outer solution I, (2) Δφ_2_ between glass and inner electrolyte II, (3) Δφ_3_ at the inner reference electrode, (4) Δφ_4_ at the external reference electrode, (5) the diffusion potential at both reference electrodes, *E*_d_ = Δφ_d,I_ + Δφ_d,II_, which is caused by small amounts of various ions passing the diaphragm in both directions with different velocity, driven by concentration gradients between the adjacent solutions. Even at the same *p*H of inner electrolyte and outer test solution, the measured chain voltage does not equal zero, because the slightly different soaking layers on both glass sides cause an instable *asymmetry* voltage *E*_as_ = Δφ_as_. Due to the dissociation of functional groups at the glass surface, the slope of the *E* vs. pH function may be smaller than the theoretical Nernst response, d*E/*d*p*H = ln 10 *·* (*RT*/*F*) ≈ 0.059, at 25 °C.

The practical chain zero-point, *U* = 0 V, in commercial glass electrodes is at about pH 6.84, and drifts to slightly higher pH values the more the membrane glass corrodes in the inner electrolyte. The offset voltage at pH 7 is determined mainly by the diffusion potential in the test solution. The intersection point of the isotherms (pH_iso_|*U*_iso_) in Figure 1-c is calculated by means of the pH measurements in two buffer solutions (A and B) at two different temperatures (1 and 2):

(3a) $$U_{\text{iso}}=\frac{U_{\text{B}1}S_{2}-U_{B2}S_{1}-S_{1}S_{2}(p{\text{H}}_{B2}-p{\text{H}}_{\text{A}2})}{S_{2}-S_{1}};S_{1}=\frac{U_{B1}-U_{A1}}{p{\text{H}}_{B1}-p{\text{H}}_{A1}}$$

(3b) $$p{\text{H}}_{\text{iso}}=\frac{U_{\text{iso}}-U_{A1}}{S_{1}}-p{\text{H}}_{A1};S_{2}=\frac{U_{B2}-U_{A2}}{p{\text{H}}_{B2}-p{\text{H}}_{A2}}$$

Hence the terminal voltage of a pH meter is not absolutely defined; it must be calibrated against standardized pH *buffer solutions* [7]; e.g. hydrochloric acid (0.1 mol/L, pH 1.094), potassium hydrogen phthalate (0.05 mol kg^–1^, pH 4.005), Na_2_HPO_4_/KH_2_PO_4_ (each 0.025 mol kg^–1^, pH 6.865), sodium tetraborate (0.01 mol kg^–1^, pH 9.180), NaHCO_3_/Na_2_CO_3_ (each 0.025 mol kg^–1^, pH 10.012) at 25 °C.

Solutions of strong acids, such as 0.001 molar HCl (pH 3.00), exhibit nearby no *temperature dependence* of pH, because the H^+^ concentration is determined by the dissociation of the acid. In alkaline solutions, however, the pH value is determined by the autoprotolysis of water, and decreases with rising temperature; e.g. 0.001 molar NaOH: pH 11.94 at 0 °C; 11.00 (25 °C); 10.26 (50 °C).

The *cross-sensitivity* of the pH electrode is described by the Nikolsky-Eisenman equation, containing the selectivity coefficients *k_i_* for all other single charged ions present [8,9]:

(4) $$E=E_{0}+\frac{RT}{F}\ln\left(a_{H^{+}}+k_{1}a_{\text{Na}+}+k_{2}a_{{\text{K}}^{+}}+\ldots \right)$$

The cross-sensitivity is measured in different solutions after adding rising amounts of NaClO_4_, e.g., 0, 0.01, 0.1, and 1 mol/L, respectively. Useful solutions for this purpose are [10]:

a) 2,2-Bis(hydroxyethyl)amino-tris(hydroxymethyl)methane, 20.924 g/L, in HCl, 0.05 mol/L; pH 6.58
b) Trishydroxymethylamine, 18.17 g/L, in HCl, 0.1 mol/L; pH 7.90
c) Ethanolamine, 3.054 g/L, in HCl, 0.03 mol/L; pH 9.39
d) Piperidine, 10,644 g/L, in HCl, 0.1 mol/L; pH 11.34
e,f,g) Tetramethylammoniumhydroxide, 25% (54,69 g/L, 105.73 g/L or 328,14 g/L, respectively), in HCl, 0.1 mol/L, exhibits pH 12.61, pH 13,3, and pH 14.0 respectively. NaCl is added to this solution.

### 2.2. Absolute Measurement of pH in Dilute Aqueous Solution: IUPAC Recommendation

The Harned cell [11] is a primary method of measurement [12] in order to incorporate pH determinations into the SI system, based on a well-defined measurement equation in which all of the variables can be determined experimentally in terms of SI units. The Harned cell, a cell without transference, defined by (–) Pt|H_2_|solution, KCl|AgCl|Ag (+), consists of a hydrogen electrode (dry hydrogen at atmospheric pressure *p*) and a silver-silver chloride electrode, and exhibits no diffusion potential, which usually occurs at any two adjacent liquid phases. The measurement comprises three steps:

1. The cell (–) Pt|H_2_|HCl(*m*)|AgCl|Ag (+) is filled with hydrochloric acid (e.g. *m*_HCl_ = 0.01 mol kg^–1^, γ_±HCl_ = 0.904 at 25 °C), and the potential difference according to the spontaneous cell reaction, ½H_2_ + AgCl → Ag_(s)_ + H^+^ + Cl^–^ is measured. From this *E*^0^ is calculated (*p*^0^ = 101 325 Pa):
  (5) $$\Delta E_{1}=E_{KC1|\text{AgC}1|Ag}^{0}-\frac{RT\cdot \ln10}{F}\mathrm{lg}\frac{{\left(m_{HC1}\cdot \gamma_{\pm HC1}\right)}^{2}}{\sqrt{p/p^{0}}\cdot {\left(1{\text{molkg}}^{-1}\right)}^{2}}$$
2. The Harned cell is then filled with a test solution, and the acidity function *p*(*a*_H_γ_Cl_) = –lg(*a*_H_γ_H_) is measured for at least three molalities of added potassium chloride (*I* < 0.1 mol kg^–1^). *p*(*a*_H_γ_Cl_)^0^ is found by linear extrapolation towards infinite dilution. In practice, the cells of step 1 and step 2 are operated simultaneously in a thermostat bath at 25 °C, so that Δ*E*_2_–Δ*E*_1_ is independent of the standard potential difference and the assumption that the standard hydrogen potential equals *E*^0^(H^+^|H_2_) = 0 at all temperatures:
  (6a) $$\Delta E_{2}=E_{KC1|\text{AgC}1|Ag}^{0}-\frac{RT\cdot \ln10}{F}\mathrm{lg}\frac{m_{\text{H}}m_{\text{Cl}}\cdot \gamma_{\text{H}}\gamma_{\text{Cl}}}{\sqrt{p/p^{0}}\cdot {\left(1{\text{molkg}}^{-1}\right)}^{2}}\Rightarrow$$
  (6b) $$p\text{H}=\underset{m\text{C}1\to 0}{\lim}-lg(a_{H}\gamma_{\text{C}1})=\underset{mC1\to 0}{\lim}\frac{\Delta E_{2}-E_{\text{KC}1|\text{AgC}1|\text{A}g}^{0}}{(RT/F)\cdot \ln10}+\mathrm{lg}\frac{m_{\text{Cl}}}{\left(1{\text{molkg}}^{-1}\right)}-\mathrm{lg}\gamma_{C1}$$
3. The pH value in Equation (6b) is calculated according to the Bates-Guggenheim convention that the immeasurable activity coefficient of the chloride ion γ_Cl_ is estimated by help of the Debye-Hückel theory. For KCl (*z*_+_ = *z*_–_ = 1), the ionic strength *I*, and the molar concentration *c* (in mol/L), or molality *m* (in mol kg^–1^), respectively, are identical:
  (7) $$\mathrm{lg}\gamma_{C1}=\frac{-A\sqrt{I}}{1+B\sqrt{I}},\text{and}A=0.509(at25{}^{\circ}C);B=1.5(at5-50{}^{\circ}C);I=\frac{1}{2}\sum_{i}Z_{i}^{2}C_{i}$$

For *non-aqueous solvents* [13], having an water-like autoprotolysis, LH + LH ⇌ [LH_2_]^+^ + L^–^, the ⇌ neutral point, similar to pH 7 in pure water, is defined by $n=-\mathrm{lg}\sqrt{K}$, wherein *K* is the equilibrium constant of the autoprotolysis. Such a specific *p*H*_n_* scale for each solvent does not allow the direct comparison with aqueous solutions.

A universal scale of acidity for all media must be based on the activity of free protons, *p*H = –lg *a*_H_ [14], and can be measured relative to a standard hydrogen electrode in this medium as shown above. For concentrated sulfuric acid (pH = −10), and saturated KOH solution (pH 19), the usual range between pH 0 and 14 is reached again by dilution.

### 2.3. Solid State pH Electrodes

The fabrication of an all-solid-state glass electrode [15] is complicated by the internal reference electrodes, which do not work precisely in the absence of a buffer solution. Trials to coat platinum wires with glass were not successful. Silver layers, amalgams (of sodium, lithium or cadmium), metal alloys and tungsten bronzes on the inner porous glass membrane are known (see Section 7).

*Ion Selective Field Effect Transistors* (ISFET) have been developed since the 1970s [16], but have not yet reached the precision of the pH glass electrode. ISFET pH sensors are limited to a pressure of about 2 bar and temperatures up to about 85 °C.

**Figure 2.**
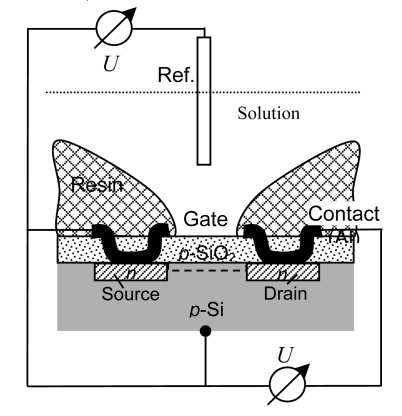
Principle of a solid state pH electrode (ISFET). The SiO_2_ layer at the gate is covered by an ionsensitive layer. The gate voltage *U*_G_ is the potential difference between reference electrode and *n*-channel (dashed line between source and drain).

Basically, the current flowing between two semiconductor electrodes (drain and source) is controlled by the electrostatic field generated by the protonated third electrode (gate), which is placed between drain and source. Usually the gate is coated by (i) a ceramic material such as Al_2_O_3_, Si_3_N_4_, Ta_2_O_5_, ZrO_2_, GaN (Table 1); (ii) an organic material, e.g. valinomycin in a resin; (iii) a polymer such as PTFE, polypyrrol or polyethylene naphthalate (PEN), or (iv) a catalytical metal layer, e.g. Pt. Instead of measuring the potential difference on two sides of a glass membrane, the current flowing through the transistor (MOSFET) is observed. For practical measurements in liquids, the electrical circuit must be closed with a reference electrode (Figure 2). On any change of pH value and thus the gate potential, the voltage supply to the reference electrode is re-adjusted by electronic feedback in order to keep the measured drain current constant to a predefined value – usually the current at the isothermal point in order to avoid the influence of temperature. Highly accurate amperometers are required (< 1 μA) to measure the drain-source current, while the gate voltage is increased.

**Figure 3.**
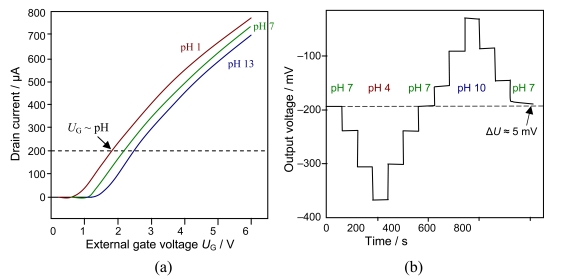

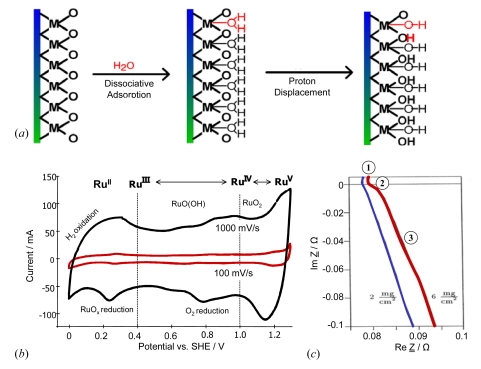
(a) ISFET transfer characteristics at different pH at 25 °C. The gate is coated with a RuO_2_ thin film. (b) Hysteresis widths during the pH 7–4–7–10–7 loop cycle [19]. (a) Dissociative adsorption of water at platinum metal oxides and proton conduc- tivity [38]: [Ru]OH_2_ ⇌ [Ru]OH^−^ + H^+^ ⇌ [Ru]O^2−^ + 2H^+^. (b) Cyclic voltammogram of a RuO_2_ electrode in sulfuric acid (3 mol/L) at different scan rates. Standard potentials: Ru^3+^/Ru^2+^ (0.24 V), Ru^IV^/Ru^3+^ (0.86 V), Ru^IV^/RuO_4_ (1.4 V). (c) Increase of *ac* impedance of a cell of two RuO_2_/Ni electrodes in potassium hydroxide solution with rising oxide coating: 1 = electrolyte resistance, 2 = grain boundary resistance, 3 = diffusion impedance.

pH-sensitive films of RuO_2_ can be sputtered or screen-printed on silicon, alumina, pyrex, or polyester foil (Figure 3-a). The electrochemical sensor exhibits three basically nonideal characteristics:

- *Drift effect*: The slow nonrandom change of output voltage with time (by some mV h^–1^) in a solution with constant composition and temperature. Measurement circuit, reference electrode, and device body are greatly affected.
- *Hysteresis or memory effect*: When the ISFET is measured many times in the same pH buffer solution, different output voltages at the buffer solution–insulator interface occur. This apparent delay of the pH response creates a loop cycle with different hysteresis widths in different pH buffer solutions (Figure 3-b).
- *Optical effect:* The output voltage of the ISFET changes (by some ten mV) when the light is switched on and off. For surface potential mapping, on the dry backside of the silicon substrate, a light-emitting diode may be applied to generate a photocurrent, the size being a measure of the surface potential at that particular region.

### 2.4. Enamel Electrode

A pH-sensitive enamel zone, deposited on a sturdy steel tube, can be used for pH measurements under mechanical stress [20]. A flat steel diaphragm, at the end of the tube, is connected to the reference electrode. The probe has an individual slope around 55 mV pH^–1^; the working range is between pH 0 and pH 10. The sodium error, especially at elevated temperatures, limits applications to pH 6–8 at temperatures above 140 °C. pH_iso_ (see Figure 1-c) lies around pH 2.

The composition of a glass having a similar thermal expansion coefficient than steel was reported as (by weight): 68.61% SiO_2_, 18.41% Na_2_O, 6.44% MgO, 6.54% UO_2_ [2]. Mixed electronically and ionically conducting glasses in order to connect two pH glass layers, may contain iron oxide; such as 36.9% SiO_2_, 16.1% Na_2_O, 43.4% Fe_2_O_3_, 2.6% Al_2_O_3_ [2].

### 2.5. Gel Membrane Electrodes

Proton-sensing compounds – such as *N*-octadecylmorpholine – in a polymer matrix [21] are suitable for pH measurements in a narrow pH range [22].

### 2.6. Electrochemically Active Monolayers

Redox-active groups in a receptor adsorbate on a conducting substrate may indicate pH changes. Thioethers adsorb strongly on a gold surface through the sulfur atom; the alkyl groups may be linked to a ferrocene group and a carboxyl acid group, e.g. (C_5_H_5_)Fe(C_5_H_4_)–(CH_2_)_6_–S–(CH_2_)_7_COOH. On a change from pH 6.5 to pH 1, the redox peak of ferrocene in the cyclic voltamogram is shifted by about 200 mV [23]. Cyclodextrins, calixarenes, and cavitands are able to bind organic guest molecules in a hydrophobic cavity, which can be used for biosensors.

### 2.7. Colorimetric Sensors and Optodes

Advantages and disadvantages of different pH monitoring methods are compiled in Table 2. The use of optical sensors for spectroscopy and light scattering measurements is restricted to nearby colorless solutions without substantial changing of transparency. pH indicators require the absence of strongly oxidizing or reducing agents. For pH measurements in biological media, quinoline derivates have been reported as a fluorescence probe.

## 3. Metal Electrodes for pH Determination

A number of metal electrodes has been described in literature [24] for gas sensors that can basically be utilized for the detection of acid gases dissolved in liquids, too (Table 3).

### 3.1. Hydrogen Electrodes and Storage Electrodes

Platinum black, purged with pure hydrogen (Section 2.2), is most corrosion resistive, but it is readily poisoned by CN^–^, H_2_S, and As_2_O_3_. Platinum absorbs oxygen if not stored under hydrogen permanently, and heavy metal ions, nitrate, and nitrophenols are reduced on its surface.

Palladium, ruthenium, and osmium, if resaturated with hydrogen periodically, work as pH probes, without it being necessary to maintain them permanently in a current of hydrogen (such as platinum). It holds *E* = −0.0591 pH, or pH = −16.92·*E* (at 25 °C).

The standard potential of the half-cell reveals whether a redox system is stable in aqueous solution or works as an oxygen electrode (*E*^0^′ > 0.815 V at pH 7) or a hydrogen electrode (*E*^0^′ < −0,414 V at pH 7). For example, the redox system Cr^3+^ + e^–^⇌Cr^2+^, due to the ínstable Cr^2+^, decomposes water and is therefore a hydrogen electrode, H^+^ + e^–^⇌½H_2_, which, however, can be stabilized at pH 7.

The *quinhydrone* electrode [25] consists of an equimolar mixture of benzoquinone and 1,4-dihydro-xybenzene, which is contacted by a platinum rod. The standard redox potential *E*^0^ = – (*RT*/*F*) ln [*p*_H2_]^1/2^ ≈ +0.70 V corresponds to the low hydrogen pressure of about 2·10^–24^ bar. This quasi-hydrogen electrode is restricted to diluted acid solutions. At pH ≥ 8 it is decomposed by oxygen.


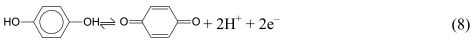


### 3.2. Metal-metal Oxide Electrodes

Metal surfaces which form unsoluble hydroxides in aqueous solution can be used for pH determinations. The redox potential of the *antimony* electrode [26, 27] – see Table 4 – depends directly on the proton activity of the solution in the range between pH 3 and pH 11:

(9) $$E=E^{0}+\frac{RT}{3F}\ln a{(\text{Sb}}^{3+})=E^{0}+\frac{RT}{3F}\ln\frac{K_{L}}{K_{\text{W}}}+\frac{RT}{F}\ln a{(\text{H}}^{+})=E^{0\prime }-\frac{RT}{0.4343\cdot F}\cdot p\text{H}$$

where *K*_w_ *= a*(H^+^)*·a*(OH^–^) denotes the ionic product of water. *K*_L_ is the solubility product of antimony hydroxide according to Sb_2_O_3_ + 3H_2_O ⇌ 2Sb(OH)_3_ ⇌ 2Sb^3+^ + 6OH^–^. The surface of antimony wires can be oxidized anodically or in a potassium nitrate melt. An immediate electrode response is achieved if Sb_2_O_3_ is added to the melt when puring the antimony electrode, too. The potential of the antimony electrode depends on the oxygen partial pressure and parasitic electrode reactions; mechanical cleaning of the electrode surface is uncritical.

The measured cell voltage of the antimony electrode against a reference electrode is disturbed by reducing and oxidizing agents in the solution – a problem of all metal electrodes. pH_iso_ of the highly asymmetric cell (see Figure 1-c) lies at a negative pH value of -3.

In alkaline solutions the *bismuth* electrode was described [28]. Approximately linear functions of potential versus pH were observed with Sn, W, Fe, Ir, Os, Ag, Cu, Zn, and other metals, mainly in oxygen-free buffer solutions. Mostly, the slope does not equal the theoretical value of (ln 10) *RT*/*F*, and is limited to a narrow pH window, and depends on the anions present in the solution. These electrodes are not simply hydrogen electrodes; several simultaneous potential-determining processes give rise to a mixed potential which may come close to the reversible hydrogen potential.

## 4. Platinum Metal Oxide Probes in Aqueous Solutions

Metal oxides are useful materials for chemical sensors that utilize changes of electric charge at electrode-electrolyte interfaces. Both electronically and ionically conducting metal oxides have been described for resistive gas sensors [29], which, however, require a separate heating layer to accelerate chemical reactions at the interface between gas space and porous oxide.

- Hydrogen-sensitive: Co_3_O_4_, ZnO, SnO_2_, MoO_3_, WO_3_, MnO_2_
- Oxygen-sensitive: TiO_2_, SrTiO_3_, BaTiO_3_, ZrO_2_, Fe_2_O_3_, CoO, ZnO, SnO_2_, La_2_O_3_

Metal oxides [30] behave as mixed electronic and ionic conductors due to their oxygen defect stoichiometry. The mechanism of pH response of metal oxides might be explained by surface phenomena at ion exchanging surface sites, and does not necessarily involve pH dependent redox transitions. With respect to a future coulometric “proton titrator”, the following section investigates the redox chemistry of hydrous ruthenium dioxide around its rest potential (∼0.8 V vs. SHE) in aqueous solution.

### 4.1. Ruthenium Dioxide: Model System of a Novel Proton Probe

Since the late 1970s mixed platinum metal oxides [31], coated on titanium supports, have been developed for dimensionally stable electrodes (DSA) for chloralkali electrolysis [32]. The RuO_2_ electrode works as a quasi-hydrogen reference electrode (cf. Section 3), and is sensitive for dissolved oxygen. First, in the 1980s, oxygen intercalation [33] was assumed, with a proton activity in the liquid phase, and an oxygen activity in the solid phase: RuO_2_ + 2*z* (H^+^ + e^–^)⇌RuO_2–_*_z_* + *z*H_2_O. However, platinum oxide surfaces were found to be able to exchange protons with aqueous solutions [34]. The mechanism in Equation 10 was found, among others, by investigating the point of zero charge and the exchange of tritium ions on the inner surface of the porous material [35,36]:

(10) $$RuO_{x}{\left(OH\right)}_{y}+ze^{-}+zH^{+}⇌RuO_{x-z}{\left(OH\right)}_{y+z}$$

(11) $$\text{Simplified}:Ru^{IV}O_{2}+e^{-}+H^{+}⇌Ru^{\text{III}}O(OH)$$

The pseudocapacitance C(*U*) = d*Q*/d*U* of the RuO_2_ electrode [37] arises from kinetically inhibited redox processes at the metal oxide-liquid interface, and depends strongly on frequency, temperature, and the applied voltage *U*.

The Helmholtz double-layer capacitance is always superimposed by faradaic delivered charges *Q* from the battery-like redox steps involved in the potential-determining charge-transfer reaction across the interface. Water molecules saturate the free valences in the disturbed rutile lattice. By dissociative adsorption of water – as shown in Figure 3-a – the RuO_2_ surface is covered by hydroxide groups, which try to form oxide sites by the release of protons. The process might be driven by the goal to compensate the oxygen defect stoichiometry of the oxide.

The *cyclic voltammogram* in Figure 3b shows the overlapping, highly reversible, redox processes of Ru(IV), a small amount of Ru(III) and other species. The thermodynamically calculated standard potential of the redox reaction 2RuO_2_ +2H^+^ +2e^−^ ⇌ Ru_2_O_3_ + H_2_O at *E*^0^ = 0.937 V [39] corresponds to a current peak in the cyclic voltammogram, which arises during the precipitation reaction according to: 2RuCl_3_ + 6KOH + ½O_2_ → 2(RuO_2_·1.5H_2_O) + 6KCl [40].

Tetravalent ruthenium, e.g. in K_2_Ru(OH)Cl_5_, can be reduced to Ru(III) with *hydrogen*; and “Ru(OH)_3_” can be oxidized in the *air* to RuO_2_·2H_2_O. The peak currents, *I* = *C v*, in the oxidation wave at about 0.7–0.9 V in Figure 3-b increase if oxygen is blown on the RuO_2_ electrode. Solutions of RuCl_3_·3H_2_O, which are reduced electrochemically to pink [Ru(H_2_O)_6_]^2+^ ions, are immedeately oxidized back to the yellow [Ru(H_2_O)_6_]^3+^ by oxygen, or less fast by the decomposition of water. Ru(II), with hydrogen bound side-on, is known as a proton source, [Ru(H_2_O)_5_(H_2_)]^2+^→[Ru(H_2_O)_5_H]^+^ +H^+^.

A RuO_2_ film that is electrochemically oxidized on a quartz crystal microbalance loses a mass of 56.3 u per electronic charge, which corresponds roughly to [H(H_2_O)_3_]^+^ (mass 55) [41], cf. Equation 11. To explain the ionic conductivity of RuO_2_, a bulk diffusion process including H_3_O^+^ species was suggested [42]; later the low activation energy of 4–5 kJ mol^−1^ was attributed to a Grotthus-type *proton hopping* mechanism. The protons (or hydroxide sites), formed by dissociative adsorption of water, can penetrate into the porous electrode material. The impedance spectrum in Figure 3-c shows a diffusion branch at low frequencies which depends clearly on the thickness of the active layer.

The voltammetric charge *Q* increases with rising RuO_2_ mass until the film gets too thick; solid-phase redox reactions in the bulk material contribute by less than 10% to the overall capacitance. In particles larger than 30 nm, therefore, most of the charge capability will remain unused in the particle core.

#### pH sensitivity

According to the Nernst equation, the redox potential at the RuO_2_ electrode (Equation 11) depends on the pH. At 25 °C, and nearby equal activities of Ru(III) and Ru(IV), which approach *a* = 1 in the solid state, the electrode reduction potential therefore drops in alkaline solution:

(12) $$E=E^{0}-\frac{RT}{F}\ln\frac{c({\text{Ru}}^{\text{III}})}{c({\text{Ru}}^{VI})\cdot c({\text{H}}^{+})}=E^{0}-\frac{\ln10\cdot RT}{F}\left(\text{pH}+\log\frac{c({\text{Ru}}^{\text{III}})}{c({\text{Ru}}^{VI})}\right)$$

(13) $$\text{At}25{}^{\circ}\text{C approximately}:E=E^{0}-0,059\cdot p\text{H}$$

In practice, the theoretical slope of −59 mV/pH is not reached. Obviously, the surface groups can additionally take up or release protons without electron transfer (see Figure 3-a). If RuO_2_ electrodes are soaked in diluted HCl (< pH 2), the Nernst slope can be improved. Actually, Ru(IV) in aqueous solution was shown to be cluster ions of the type H*_n_*[Ru_4_O_6_(H_2_O)_12_]^(4+^*^n^*^)+^ [43].

**Figure 4.**
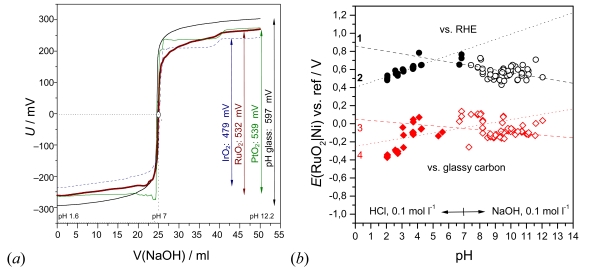
(a) Quasi-stationary potentiometric titration curve of 0.025 molar hydrochloric acid with 0.1 molar sodium hydroxide solution at different metal oxide electrodes (hydrous RuO_2_ by alkaline precipitation, bound in alkyd resin; thermal decomposition of H_2_IrCl_6_, and H_2_PtCl_6_ on a nickel support). Counter electrode: glassy carbon. For the purpose of comparison, the curves are shifted into the voltage range of a commercial glass electrode. (b) Stationary long-time test of a RuO_2_|Ni electrode in tap water with different reference electrodes. Counter electrode: glassy carbon; average temperature 23 °C. Each data point was measured for 24 hours after adding small amounts of acid or base. For comparison, Nernst slopes: 1 = –(0.059/2); 2 = +0.059; 3 = –(0.059/4); 4 = +(0.059/2).

Performance and challenges of RuO_2_|Ni electrodes are shown in Figure 4. A 30 μm thick hydrous RuO_2_ layer (1.7 mg cm^–2^, bound in alkyd resin) on a nickel support, versus a glassy carbon counter/reference electrode, works as well as a glass electrode during potentiometric acid-base titrations. The voltage jump between pH 1.6 and 12.2 equals 532 mV, in contrast to: plain nickel sheet (176 mV), alkyd resin on nickel (346 mV), a cell of two identical RuO_2_/Ni electrodes (∼100 mV, no S-shape in Figure 4-a) and two glassy carbon electrodes (∼200 mV, no S-shape). During aging of the sensor, the potential difference between pH 0 and 14 drops slowly, but the endpoint is displayed correctly. The stability during long-term measurements up to 200 h at different pH values is good (about ± 20 mV).

#### Consideration of adsorbed gases

The stationary application as a pH probe in tap water (Figure 4-b) is complicated by dissolved oxygen and metal ions, and opposed potential determining processes at the working electrode and the counter/reference electrode. In diluted acids and bases, and, as well, in commercial pH buffer solutions, the RuO_2_/Ni and PtO_2_/Ni electrodes (vs. glassy carbon and RHE) do not clearly indicate the linear trend of increasing pH values. Cell voltage increases from pH 0 to 7, but decreases from pH 8 to 14. The nickel support can be neglected if the metal oxide layer is thick enough (> 100 μm); the cyclic voltamogram shows the metal oxide surface only. As the pH response is rather complicated by more or less unknown Ru cluster ions, a simplifying formal approach based on absorbed gases is tried.

The RuO_2_ negative electrode behaves like a quasi-reversible hydrogen electrode, and the RuO_2_ positive electrode behaves like a quasi-reversible oxygen electrode in aqueous solution. Actually, at the potential of zero charge φ_z_ the adsorbed water molecules change their orientation at the electrode surface: from the adsorption with the H atoms (φ < φ_z_) to the adsorption with the O-atoms (φ > φ_z_).

1. In acid solution, as well as it is known for the hydrogen oxidation at a hydrogen electrode, the electrode potential increases with rising pH. Protons are released by the dissociative adsorption of water and superacid OH groups. Simplified, by the help of rutile lattice sites [Ru], the potential determining surface process at the more negatively charged RuO_2_ electrode reads:
  (14) $${\left[Ru^{\text{III}}\right]}_{2}\text{O}+{\text{H}}_{2}O⇌2[Ru^{\text{III}}]O\text{H}\mathrm{or}{\left[\text{RuO}\right]}_{2}{\left(H\right)}_{2}⇌2[Ru^{\text{IV}}]O+2H^{+}2{\text{e}}^{-}$$
  (15) $$\text{Formally}:[\text{Ru}]{\text{H}}_{2}⇌[\text{Ru}]+2{\text{H}}^{+}+2{\text{e}}^{-}$$
2. In alkaline solution, as well as it is know for the oxygen reduction at an oxygen electrode, the electrode potential decreases with rising pH. By the dissociative adsorption of water, hydroxide sites are formed and bound in ruthenium cluster ions.
  (16) $$4[Ru^{IV}]O\mathrm{or}2{\left[Ru\right]}_{2}(O_{2})+2H_{2}O+4e^{-}⇌[Ru]+4OH^{-}$$
  (17) $$\text{Formally}:[Ru]O_{2}+4e^{-}+2H_{2}O⇌[Ru]+4OH^{-}$$

With respect to Equations (15) and (17), RuO_2_ can be considered as a fuel cell sensor, especially as adsorbed hydrogen and oxygen recombine to water. However, the recombination is kinetically inhibited. The relative predominance of dissolved oxygen and hydrogen in water, within the electrochemical stability window of water, can be estimated by the formal reaction (18) at 25 °C [44]:

(18) $$H_{2}+2H_{2}O⇌O_{2}+6H^{+}+6e^{-}$$

(19) $$E=0.819-0.0591\cdot \text{pH}+0.0098\log\frac{p({\text{O}}_{2})}{p({\text{H}}_{2})}$$

At potentials below *E*, dissolved hydrogen is thermodynamically stable in aqueous solutions; at potentials above *E* oxygen is predominant. In Figure 4-b, line 1 (pH > 7) corresponds therefore to absorbed oxygen, and line 2 (pH < 7) to absorbed hydrogen, which determine the cell voltage of the metal oxide-glassy carbon cell.

Recently, a mechanistic study and ^18^O labeling experiment on the photochemical oxidation of water at binuclear ruthenium complexes [45] illustrated the four oxidative electron-transfer process that takes the catalyst from its initial II,II oxidation state up to the formal IV,IV oxidation state. Once the Ru(IV) oxidation state is reached, two additional slower kinetic processes take place, corresponding to the formation of an intermediate that finally evolves oxygen. This result clarifies the intramolecular reaction pathway for the formation of the oxygen−oxygen bond in the case of adjacent ruthenium(IV) atoms.


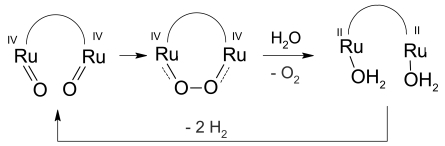


#### Cross sensitivity

The proton exchange mechanism at a glass surface can basically be reproduced by platinum metal oxide-hydrates. However, the RuO_2_-solution interface appears to be not a selective proton conductor, because its conductivity depends considerably on the ionic composition of the solution. In distilled water and phthalate buffer (pH 7), quite different electrode potentials are measured at the metal oxide electrode due to the difference of *ionic strength*. Further problems are caused by the formation of ruthenium cluster ions, the sensitivity against other ions than H^+^, and the type of the reference electrode.

The Nernst slope, which generally deviates from 59 mV pH^–1^ (25 °C), is nearly independent of dissolved anions in the solution (such as 0.1 mol/L of SO_4_^2–^, Br^–^, Cl^–^, and NO_3_^–^). However, commercial RuO_2_ resistive pastes, which contain PbO, exhibit a slope which depends on different anions significantly [46].

Reducing agents (e.g. ascorbic acid, Fe^2+^, sulfite) and oxidants (H_2_O_2_, I^–^) damage the reversibility at both anodic and cathodic potentials, which reveals the role of adsorbed hydrogen and oxygen for the measured mixed potentials. Traces of platinum (being a recombination catalyst) significantly alter the cyclic voltammogram of a RuO_2_ electrode, especially in the hydrogen region.

#### Preparation

Since the 1980s, etched titanium sheets have been repeatedly dip-coated in an alcoholic solution of RuCl_3_·3H_2_O, followed by drying and pyrolysis at 300–350 °C [47]. Higher decomposition temperatures destroy the active surface area of the electrode, and yield less marked jumps of cell voltage in the acid-base titration curve at pH 7.

In the 1990s, it got evident that the specific capacitance at the electrode-electrolyte interface can be enhanced by a residual amount of water in the RuO_2_·*x*H_2_O material [48], which corresponds to the presence of Ru(III) in the disturbed rutile lattice. High surface area ruthenium oxide-hydrate; RuO_2_·*x*H_2_O [49], prepared by alkaline precipitation [50] from RuCl_3_ solutions at about pH 7.5 (sol-gel process [51]), washed several times with water, dried at 90 °C, and finely dispersed in a mixture of polyalcohols, can be screen-printed on carbon fiber paper or nickel supports [52]. Commercial RuO_2_·*x*H_2_O has a water content between *x* = 1 and 3. Heat treating at 200 °C reduces the water content to *x* = 0.4, or 5% by weight.

The IR absorption of solid RuO_2_ powders, due to the stretch vibration of H-bridged OH-groups above 3000 cm^−1^, is strongest for sol-gel RuO_2_, whereas the thermally prepared powders contain less adsorbed or chemically bound water. In contrast to the “thermal” powders, the colloidal sol-gel RuO_2_ forms a considerable amount of coloured ions in aqueous solution, which appear to be important for redox capacitance. The SIMS spectra of sol-gel RuO_2_ reveal mass peaks at 133 u and 149 u, which might be attributed to the predominant Ru(III) and Ru(IV) species minus a proton, and cannot be found in single crystalline RuO_2_ in this significant amount [53]. RuO_2_ electrodes under current age by partial oxidation of the surface sites, i.e. by a loss of Ru(III) surface sites, which are essential for the dissociative adsorption of water involving proton conductivity.

#### Non-aqueous solutions

The interfacial capacitance of platinum metal oxides in organic solutions is considerably lower than in aqueous solution. RuO_2_ typically contains a certain amount of residual water, which allows the development of an equilibrium potential based on the dissociative adsorption of water in non-aqueous solutions, too. Comprehensive information on this topic is not available in the literature.

### 4.2. Applications of Ruthenium Dioxide Sensors

Usual thick film sensors are prepared by three inks or pastes which are screen-printed onto alumina: Ag-Pd paste as conductor, metal oxide paste as active surface and an overglace paste as protector. In recent years, finely dispersed platinum metal oxides were coated on carbon particles or bound in polymers [54,55,56]. Polymer bound metal oxide electrodes can be fabricated by coating metal plates with a thin layer of a mixture of metal oxide powder in commercial varnish based resins. The best pH sensitivity is achieved by a mixture of 10% sol-gel RuO_2_·*x*H_2_O (water content ∼7.2%) in a matrix of epoxy or polyester [57]. Reactive sputtering of platinum metal targets in argon-oxygen atmospheres is used to produce 1 μm thick oxide electrodes on alumina and silicon substrates; palladium and platinum oxides were found to be less stable than ruthenium oxide [58]. An overview of applications [59] is given in Table 5. Both potentiometric and amperometric sensors [60] have been used.

#### ***Biosensors*** [69,70,71]

**Figure 5.**
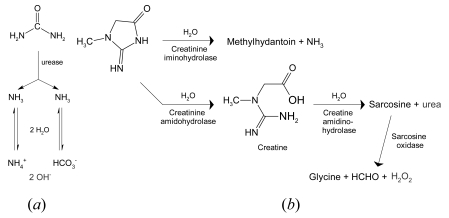
(a) Principle of the urea biosensor based on pH-measurements. By enzymatic hydrolysis, alkaline products are formed. (b) The creatinine sensor is based on the detection of consumed oxygen or produced hydrogen peroxide during the enzymatic conversion of the analyte.

Among a large variety of potentiometric sensors using biocatalytic and bioaffinity-based mechanisms, the detection of urea and creatinine is most advanced. Sensors based on RuO_2_ and urease are in development for the determination of heavy metals, which inhibit the enzymatic hydrolysis of urea: NH_2_CONH_2_ + 3H_2_O → 2NH_4_^+^ + HCO_3_^–^ + OH^–^ (Figure 5).

The pH sensitive RuO_2_ layer serves as a transducer for the ionic reaction products. On the RuO_2_ electrode, screen-printed on ceramic substrate, urease in polyvinylchloride is adsorbed and then immobilized in a polymer such as Nafion. The biosensor can be cleaned from heavy metals by a solution of EDTA; a constant urea concentration is applied in a TRIS buffer, and the change of electrode potential (vs. Ag|AgCl) is observed after a given time due to the decreasing rate of substance conversion in the presence of heavy metal ions [72].

1. Label-based *immunosensors* contain antibodies as analyte recognition parts. Horseradish peroxidase and alkaline phosphatase, e.g., catalyze reactions which produce electroactive products in immunoenzymatic biodevices. For example, at a polypyrrole coated screen-printed gold electrode, peroxidase may work as biocatalytic label converting *o*-phenylene-diamine into 2,3-diaminophenazine (in the presence of H_2_O_2_). Label-based analytical sensors with enzymatic, fluorescent, radiochemical, and nanoparticle markers rely on amperometric and optical detection rather than on potentiometry.
2. Label-free *bioaffinity sensors* were realized by the aid of protein coated ISFETs, which dynamically measure the release or uptake of protons by biologically active protein molecules bound on the semiconductor. Antibodies can be adsorbed on colloidal nanoparticles (Au, Ag) in a polymer matrix (e.g. gelatin, nafion, polyvinyl butaral, thiosilane gel, poly(*o*-phenylenediamine)) on a metal support. Potentiometric genosensors – as field effect devices or membrane ion-selective electrodes, modified with oligonucleotides – shall detect complementary DNA sequences.

Although the biorecognition is mostly specific, the generation of the potentiometric signal remains unclear, and are strongly affected the composition of the analysed samples. Biosensors based on voltammetry, piezoelectricity, or optical spectroscopy seem more promising for practical applications.

### 4.3. Iridium Dioxide

IrO_2_ [73] promises to be a superior material for pH measurements in technical media, such as fuels [74], food applications [75], and in biological media [76]. Irdium oxide provides: (i) a wide linear pH response range, with negligible interference of ions and complexing agents, (ii) a fast and stable response in aqueous, nonaqueous, non-conductive, and even corrosive media, (iii) high conductivity, low temperature coefficient, and no requirement for pretreatment.

#### Redox chemistry

In air-saturated solutions, iridium is covered by adsorbed oxygen atoms, which take part in redox reactions involving hydrogen ions. Anodic oxidation or heating in an oxygen atmosphere creates a surface layer of IrO_2_ with improved redox properties. The anodic and cathodic peaks in the cyclic voltammogram of an IrO_2_ electrode are attributed to the Ir^IV^/Ir^III^ redox transition, which is accompanied by the release and uptake of protons. During potential cycling of an iridium wire, the Ir^IV^/Ir^III^ transition becomes less reversible at high scan rates, resulting in the growing IrO_2_ layer; the net cathodic current is smaller than the net anodic current:

(20) $$IrO_{x}{\left(OH\right)}_{\text{y}}+\text{z}e^{-}+zH^{+}⇌IrO_{x-z}{\left(OH\right)}_{y+z}(x\approx 2,y\ll 1,z\approx 1)$$

(21) $$\text{Simplified}:Ir^{\text{IV}}{\text{O}}_{2}+{\text{e}}^{-}+{\text{H}}^{+}⇌Ir^{\text{III}}O(OH)$$

Electrodes prepared by thermal decomposition of iridium chloride or H_2_IrCl_6_ on a titanium support, as well as sputtered IrO_2_ on stainless steel and tantalum, respond to pH with a Nernstian sensitivity of nearby 59 mV/pH; whereas anodically prepared films exhibit a super-Nernstian response between 62 and 77 mV/pH [77]. The d*U*/d*p*H slope drops with time, because the oxide hydration changes. The apparent electrode potential, obtained by extrapolating to pH 0, is close to the calculated value reported by Pourbaix for the reaction 2IrO_2_ +2H^+^ +2e^–^ ⇌ Ir_2_O_3_ + H_2_O, namely *E*^0^ = 926 mV vs. NHE (682 mV vs. SCE). However, during aging over a 60 days period, the electrode potential decreases by roughly 150 mV from the initial value for a freshly prepared electrode.

#### Preparation

IrO_2_ electrodes have been prepared by (i) thermal decomposition of iridium salts, (ii) sol–gel processes, (iii) electrochemical or thermal oxidation of iridium wires, e.g. continuous potential cycling in aerated H_2_SO_4_ solution for several hours; (iv) reactive r.f. sputtering from a metallic iridium target in an oxygen plasma, (v) pulsed-laser ablation of iridium oxide targets; (vi) anodic, cathodic or electrophoretic deposition. In a polymer matrix, e.g. in Nafion [78] or PTFE-bound graphite [79], IrO_2_ can be employed as a planar thick-film pH sensor using an interdigital structure [80] of the current collectors, e.g. of silver [81].

Potentiometric solid-state sensors are claimed to be rugged, and reference solutions are not needed [82], if the pH-sensitive working electrode is an iridium wire that has been partially oxidized to IrO_2_ (about 15 μm thick). The counter-reference electrode may be nearby pH-insensitive rhodium foil that is covered by a 5 μm thick rhodium oxide layer. The potential difference declines approximately linear in the range between pH 2 to pH 12. The slope of about −30 mV/pH is coined by the Rh/RhO_2_ electrode (–26 mV/pH), whereas the Ir/IrO_2_ electrode shows Nernstian behaviour (–58 mV/pH).

#### Cross sensitivity [83,84]

Metal cations (Fe^3+^, Fe^2+^, Pb^2+^, Cu^2+^, Ag^+^) cause a small shift of some millivolts in the potential-pH response of the IrO_2_ electrode; Ni^2+^, and dissolved oxygen cause a shift of some ten millivolts. The addition of sulfate, sulfite, borate, phosphate, and ammonia to the electrolyte causes no detrimental effects; whereas oxalate, iodide, bromide, disulfite, thiosulfate, [Fe(CN)_6_]^3–^ and [Fe(CN)_6_]^4–^ alter the characteristics of the electrode more or less slightly and irreversibly.

#### Stability and recycling [85]

IrO_2_ films can be removed from electrode supports by (i) aqua regia, (ii) anodic dissolution in 2 M H_2_SO_4_; or (iii) applying successive potentials of −3.0 V and +2.5 V (vs. Ag|AgCl) in 0.3 M Na_2_HPO_4_ solution.

## 5. Tin Dioxide and Lead Dioxide

The electronical conductor *SnO_2_* [86,87] can be deposited on indium tin oxide glass (ITO) by sputtering [88]; it shows a much narrower dynamic range than the pH glass electrode; the Nernst slope equals about −58 mV/pH (pH 2 – 12). Commercial doped SnO_2_ (49 mV/pH) works mainly as a redox electrode. The sensing area, i. e. the length of the pH-sensitive tip of the glass micro electrode, should not be less than about 4 mm^2^ in order to avoid a critical reduction of the pH response (≪ 59 mV pH^–1^). Sputtering of suitable IrO_2_ films succeeds best at 20% O_2_ gas, and a pressure of 2.7 Pa (0,02 Torr).

*PbO_2_* was recognized a pH probe already in the 1970s; it can be deposited on titanium and aluminium supports [89]. The electrode potential vs. saturated calomel (SCE) decreases linearly according to the reaction PbO_2_ + H^+^ + e^–^ → PbO(OH). Anions alter the electrode potential slightly (<5% at pH 7 in 1-millimolar solution of nitrate, hydrogencarbonate, phosphate, citrate), whereas cations (1 mmol/L of Li^+^, Na^+^, Mg^2+^, Ca^2+^, NH_4_^+^) have no adverse effect. The interference of NH_4_^+^ is marked in alkaline solutions.

## 6. Transition Metal Oxides

TiO_2_ and mixed TiO_2_/RuO_2_ [90,91], Ta_2_O_5_, WO_3_, MnO_2_ [92], RhO_2_, OsO_2_, PdO [93], molybdenum bronzes and other oxides and have been described as materials for pH sensors in literature. Most oxides are useful between pH 2 to pH 11, and show a pronounced hysteresis, i. e. the electrode potential suffers a shift if the solution changes from pH 2 to pH 12, and back to pH 2 again. RuO_2_ and OsO_2_ show good accuracy (± 2 mV). Ta_2_O_5_ behaves poor in a carbon-bound electrode (± 30 mV).

pH-sensors based on ZrO_2_ and Y_2_O_3_/ZrO_2_ (YSZ) for pH measurements under high pressure are mentioned in the literature [94,95,96].

## 7. Non-Oxidic Materials and Support Materials

Aluminiumnitride (AlN) [97] and Galliumnitride (GaN) have been described for a H^+^ ion-sensitive field-effect transistor (ISFET).

*Conducting polymers* such as polypyrrole, polyaniline, and the proton conductor Nafion were described for solid-state pH sensors.

**Figure 6.**
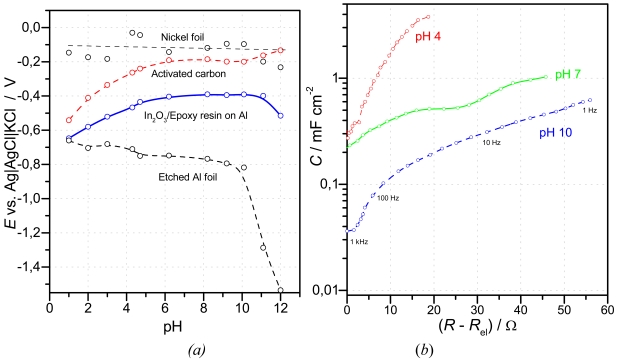
(a) Quasi-stationary pH response of plain electrodes after 10 mins at 21 °C. Size: 10x10 mm. (b) Impedance spectra of an In_2_O_3_/WO_3_ electrode (polymer-bound mixture on active carbon support) in buffer solutions at 20 °C. Reference electrode: Ag|AgCl|KCl, counter electrode: platinum [100].

*Activated carbon* and soot contain some percent of oxygen, typically bound in acidic or basic surface groups, which allow ion exchange with the surrounding solution. The quasi-stationary potential of a polymer-bound activated carbon electrode on an aluminium support, supplied by Gore for use in supercapacitors, is shown in Figure 6. Between pH 6 and pH 9, the electrode could be used as a quasi-reference electrode in aqueous solution.

*Indium oxide* in a matrix of epoxy resin on etched aluminium foil [98] extends the range of nearby constant potential range from pH 5 to pH 11. The material might be interesting for a novel reference system (see Section 7).

## 8. Reference Electrodes

The experimental conditions of the standard hydrogen electrode (SHE) [99] are all but trivial to fulfil. The most stable calomel electrode (Hg|Hg_2_Cl_2_|Cl^–^) is no longer used due to environmental concerns and the toxicity of mercury. Therefore, usually the Ag|AgCl|KCl (3.5 mol/L) reference electrode is used for chemical sensors [100,101]. The liquid filling, however, complicates miniaturisation and applications at higher pressures and temperatures.

### Silver-silver chloride electrode

The Ag|AgCl|Cl^–^ electrode works basically without any AgCl on its surface; AgCl can be dispersed in the solution, but the response to the chloride ions is slow according to the equilibrium: Ag^+^_(s)_ + Cl^–^_(s)_ ⇌ AgCl_(s)_.

**Figure 7.**
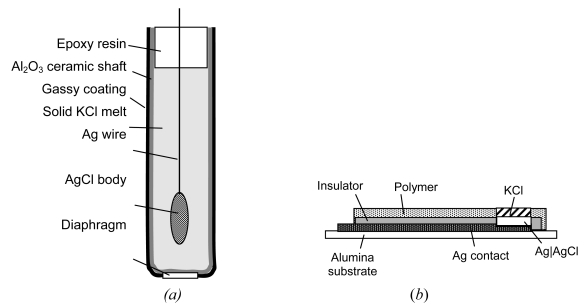
(a) Principle of an all-solid-state reference electrode after Meinsberg Kurt-Schwabe Research Institute. (b) Thick film silver-silver chloride reference electrode [102].

- At a porous AgCl coating in direct contact to the solution, redox reactions (e.g. O_2_/OH^–^) cause a mixed potential which deviates from the ideal response (+ 202 mV vs. SHE in sat. KCl solution, 20–25 °C). AgCl is a silver ion conductor. Voltage stability can be improved by small grains, and a thermal treatment of the AgCl coating below 455 °C [103]. As the chloride electrolyte is necessary, the Ag|AgCl|Cl^–^ electrode requires a liquid junction across a diaphragm. All-solid electrodes for sensors are shown in Figure 7.
- *Gel-like electrolytes* are useful at temperatures up to 140 °C and pressures up to 15 bar, however, at cost of increased diffusion potentials, irreversible bleeding, biofouling and aging of the gel. Ag|AgCl on a flat support material can be coated by a hydrogel (e.g. polyacrylamide) and surrounded by a membrane. The long-term stability of such reference electrodes in ISFETs is poor.
- *Polymer-electrolyte* reference systems (e.g. solid KCl in polyester resin) and open junctions are commercially available, e.g., under the brand name XEROLYT^®^.
- *All-solid-state* reference electrodes [104] would be most favourable. In a cylindrical shaft of porous alumina ceramic, which additionally serves as a diaphragm for the liquid junction, molten KCl is filled around a centered Ag|AgCl electrode [105]. AgCl diffuses partly in the KCl phase. Humidity from the environment provides the necessary conductivity in the hygroscopic KCl phase.
- Thickfilms of *noble metal filled glass* (e.g. Corning 015) on oxide ceramic or steel supports show increased resistivity, response times in the range of minutes, and relatively short lifetime. The reference potential does often not obey the Nernst slope. Different thermal expansion coefficients between glass and ceramic support cause cracks.
- The impact of a *glassy carbon* rod electrode under ambient conditions and the presence of dissolved oxygen, is shown in Figure 4-b. A linear function of cell voltage in the range between pH 2 to pH 12 was obtained by use of a plane gold counter-reference electrode; the total capacitance of the cell is dominated by the large capacitance of the rough RuO_2_ electrode, *C* = [*C*_RuO2_^–1^ + *C*_Au_^–1^]^–1^ ≈ *C*_RuO2_. Gold is known as an electrode material with negligible hydrogen sorption; the oxygen overpotential is higher than that of platinum.
- *Rhodium* foil, that is covered by a rhodium oxide layer, behaves nearby pH-insensitive (see Section 4.3).
- Molybdenum and tungsten *bronzes* [106], e.g. Li_0.4_Mo_0.95_W_0.05_O_3_ [107] do not significantly respond to pH changes, oxygen concentration and redox potential of the solution. However, they are sensitive to alkali ions (K^+^ < Na^+^ < Li^+^), but the preparation of single-crystalline electrodes is hardly reproducible.
- Manganese dioxide, H*_x_*MnO_2_ [108], suffers from poor reproducibility.
- Boron carbide might be useful as electrode material having a high hydrogen overvoltage.
- Prussian Blue as a reference in all-solid state pH glass electrodes was investigated in [109].

## 9. Measuring Techniques

Traditionally, the potentiometric method is favoured for pH measurements. Additionally, amperometry has been established especially for biosensors (see Table 5). The coulometric determination of proton concentrations offers for future sensor applications, e.g. based on redox active metal oxides. However, much work has to be done to finally correlate the faradaic redox reactions, which directly depend on pH, from all capacitive surface effects which reflect the electrolyte-electrode interface and the composition of the surrounding solution.

As a preliminary step to a future direct recording pH sensor, *ac* impedance spectroscopy [110] might be useful. This technique allows the separation of electrolyte resistance, charge transfer processes and diffusion processes along the grain-boundaries and in the three-dimensional pores of the material. Tungsten trioxide, in a mixture with indium oxide [100] is shown in Figure 6 for use in a capacitive pH sensor. The solution resistance *R*_el_ is subtracted from the measured impedance to exclude both the geometric dimensions of the sensor and the ionic conductivity of the solution. Then the frequency-dependent interfacial capacitance *C*_P_(ω), corrected by the solution resistance, is calculated for each frequency *f* from the measured real and imaginary parts according to Equation 22.

(22) CP(ω)=−ImZ_2πf[(ReZ_−Rel)2−(ImZ_)2]

If the *dc* resistance *R* of the pH measuring cell is assumed to be large, it holds *C*_S_(ω) = [2πf Im *Z*]^–1^ ≈ *C*_P_ at low frequencies. Then the parallel equivalent circuit *R*_el_–*C*_P_‖*R*_P_ simplifies to a series combination, of *R*_el_–*C*_S_. Both *C*_P_ and *C*_S_ are frequency-dependent differential capacitances. The pH sensor may work either at a given frequency or differential capacitance is averaged by integration in a given frequency range. The change of resistance and capacitance may also be used in commercial pH meters as a diagnostic tool for the aging of a pH electrode and the requirement for re-calibration [111].

## 10. Conclusions

1. A common reference system valid for pH measurements in all media is still missing; as well, there is no simple pH reference besides the intricate standard hydrogen electrode and the Harned cell. The pH values in non-aqueous solutions cannot simply be compared with those in aqueous systems. A coulometric proton titrator might be the solution for this problem, once appropriate directly pH dependent materials are known.
2. For the potentiometric pH determination in aqueous solutions, the glass electrode is still unsurpassed. For special applications in solutions containing fluoride or alkali, metal oxide electrodes have been introduced; whereby the antimony electrode is the most prominent example.
3. The electrode-electrolyte interface at platinum metal oxides is able to exchange protons with the surrounding solution. RuO_2_ and IrO_2_ were successfully applied for disposable applications in technical solutions and biological media. ISFETs based on platinum metal oxides suffer from poor long-term stability yet.
4. The redox pseudocapacitance of hydrous RuO_2_, in which protons are involved, is considered as a model system. Relative pH measurements based on standard buffer solutions are already possible by impedance spectroscopy. For absolute pH determination, the separation of interfacial surface charges and faradaic charges has still to be solved.
5. Platinum metal oxides can easily be coated on nickel foil by thermal decomposition of precursor solutions. Powders, e.g. obtained by sol-gel processes, can be bound in an epoxy matrix.
6. Activated carbon, glassy carbon, and possibly indium oxide seem to be useful as liquid-free reference systems in pH sensors at values between pH 5 and pH 10.

## Figures and Tables

**Table 1.** Properties of ISFET pH-sensors with different gate materials [17].

**Gate**	**Sensitivity**	**Response and stability**
SiO_2_	20–40 mV pH^–1^	non-linear response
SiO_2_ (40…110 nm, thermally grown) + Si_3_N_4_ (∼100 nm, CVD) on silicon [18]. Channel: 20 μm × 100 μm. Reference electrode: Ag|AgCl|NaCl	∼53 mV pH^–1^	Slow response; sensitivity decreases with time (formation of oxynitride)
Al_2_O_3_	53–57 mV pH^–1^	linear response, very low drift
Ta_2_O_5_	55–59 mV pH^–1^	linear response, undesired light sensitivity

**Table 2.** Specific advantages and drawbacks of different pH measuring systems.

	Range of application	Challenges
Glass electrode	Temperature: < 80…130 °C Pressure: < 60 bar (with counter pressure) Stability: ± 1 mV week^–1^	Interaction of surfactants and film formation on the glass surface in reaction mixtures Mechanical instability of the glass membrane Individual calibration of each electrode; Destruction by fluoride and highly hydroscopic solutions. Sodium error in alkaline solutions Expensive manufacture
ISFET	Temperature: < 85 °C Pressure: < 2 bar	Film formation on the surface Bad long-term stability Poor stability of the reference electrode
Antimony electrode Optical sensors	e.g. strong caustic solutions (no sodium error), fluoride containing waste water	High degree of asymmetry (pH_iso_ ≈ −3) Chloride causes potential shift Deleterious effect of sulfides, and citrates (which form complexes with Sb^III^)
	Transparent liquids Small and flexible (fiber sensors) No reference element required Signal transmittance over large distances	Change of transparency of the solution Photobleaching and wash-out of indicator phases Non-linear calibration curve with immobilized indicators

**Table 3.** Electrode reactions and useful electrode materials for the detection of gases in potentiometric sensors (voltage probe) and amperometric sensors (current probe).

	Anode reaction (electrochemical oxidation)	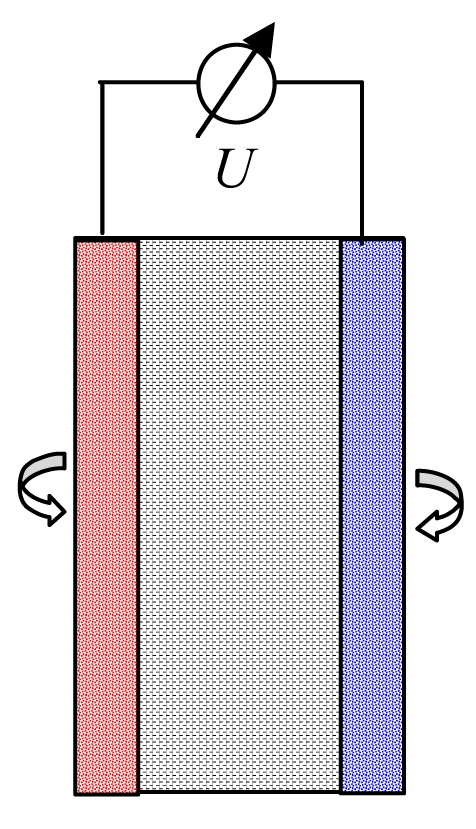	Cathode reaction (electrochemical reduction)	
Ag	2Ag + HCl ⇌ 2AgCl + H^+^ + 2 e^–^ 2Ag + H_2_S ⇌ Ag_2_S + 2H^+^ + 2e^–^ 2Ag + HCN ⇌ 2AgCN + 2H^+^ + 2e^–^		O_2_ + 4H^+^ + 4e^–^⇌2H_2_O (in acid solution)	Ag Pt
Au	SO_2_ +2H_2_O ⇌ H_2_SO_4_ + 2H^+^ + 2e^–^			
			O_2_ + 2H_2_O + 4e^–^⇌4OH^–^ (in alkaline solution)	Au C
Pt	CO + H_2_O ⇌ CO_2_ + 2H^+^ + 2e^–^			
	2H_2_O ⇌ O_2_ + 4H^+^ + 4e^–^		Cl_2_ + 2e^–^⇌2Cl^–^	Au

**Table 4.** Metal-metal oxide electrodes with pH dependent potentials. Metals having a standard potential *E*^0^ < 0 V dissolve in aqueous solution. Values in parentheses denote unstable oxides at these conditions. *) In alkaline solutions, hydroxides are existent.

Group	Material	Redox equilibrium: Ox + *z*e^–^⇌Red	*E*^0^ / V (pH 0)	*E*^0^′ / V (pH 14)	
IVa	Tin	SnO_2_ + 4H^+^ + 4e^–^⇌Sn + 2H_2_O	–0.117	–0.945	
	Lead	HPbO_2_^–^ + H_2_O + 2e^–^⇌Pb + 3OH^–^	(–0.36)	–0.537	
Va	Arsenic	As_2_O_3_ + 6H^+^ + 6e^–^⇌2As + 3H_2_O	+0.234	–0.68	
	Antimony	Sb_2_O_3_ + 6H^+^ + 6e^–^⇌2Sb + 3H_2_O	+0.152	–0.639	
	Bismuth	Bi_2_O_3_ + 3H_2_O + 6e^–^⇌2Bi + 6OH^–^	+0.317	–0.46	*)
Ib	Copper	Cu_2_O + H_2_O + 2e^–^⇌2Cu + 2OH^–^	(+0.34)	–0.36	*)
	Silver	Ag_2_O + H_2_O + 2e^–^⇌2Ag + 2OH^–^	(+0.80)	+0.342	
	Gold	H_2_AuO_3_^–^ + H_2_O + 3e^–^⇌Au + 4OH^–^	+1.50	+0.70	
IIb	Zinc	ZnO + H_2_O + 2e^–^⇌Zn + 2OH^–^	(–0.497)	(–1.260)	*)
	Mercury	HgO + H_2_O + 2e^–^⇌Hg + 2OH^–^	+0.860	+0.098	
Vb	Tantalum	Ta_2_O_5_ + 10H^+^ + 10e^–^⇌2Ta + 5H_2_O	–0.750	–1.578	
VIb	Tungsten	WO_2_ + 4H^+^ + 4e^–^⇌W + 2H_2_O	–0.119	(–0.946)	
VIIb	Rhenium	Re_2_O_3_ + 6H^+^ + 6e^–^⇌2Re+ 3H_2_O	+0.227	–0.600	
VIIIb	Iron	Fe_3_O_4_ + 8H^+^ + 8e^–^⇌3Fe + 4H_2_O	(–0.085)	–0.912	*)
	Nickel	NiO + 2H^+^ + 2e^–^⇌Ni + H_2_O	(+0.110)	–0.717	*)
	Osmium	OsO_4_ + 8H^+^ + 8e^–^⇌Os + 4H_2_O	+0.838	(≈ 0.00)	
	Rhodium	RhOH^2+^ + H^+^ + 3e^–^⇌Rh + H_2_O	+0.83	≈ 0.00	
	Iridium	Ir_2_O_3_ + 3H_2_O + 6e^–^⇌2Ir + 6OH^–^	+0.923	+0.098	
	Platinum	PtO_2_ + 4H^+^ + 4e^–^⇌Pt + 2H_2_O	+1.0	+0.14	

**Table 5.** Examples for pH sensors and measuring techniques based on RuO_2_.

Construction of the sensor: WE = working electrode, RE = reference, CE = counter	Applications and properties	Ref.
*Film layers* Screen-printed layers of graphite-based conducting inks containing 10% RuO_2_	Lemonades, wine and milk.Sensitivity: −51 mV pH^–1^Response time: < 5 min	[61]
Planar thick-film of RuO_2_·*x*H_2_O in a polymer matrix on a current collector on a alumina substrate Thick-film fabricated chemical sensor: RuO_2_ in a polymer binder on gold back contact. 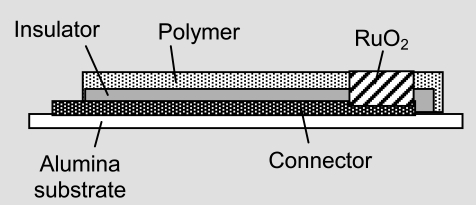	651 mV vs. Ag|AgCl (pH 0)–52 mV pH^–1^ (pH 2–10)	[62]
	Application in water-based inks:Sensitivity: −47 mV pH^–1^ (pH 4–10)pH sensitivity drift: 50 μV pH^–1^ d^–1^Previous calibration is needed.Drift of thick-film Ag|AgCl reference electrode: d*U*/d*p*H = [−0.070 ln (*t*/d) + 0.125] mV	[63]
*ISFET* RuO_2_ sensing membrane on a *p*-type silicon wafer substrate by radio frequency sputtering (Ru metal, 1.3 Pa, in Ar/O_2_; 10 W, 13.56 MHz). Drain-source voltage 0.2 V; gate voltage *U*_G_ = 0–6 V; while the drain-source current *I*_DS_ is measured.	Applications: lemonades, vinegar, milk, water.Sensitivity: ∼57 ± 1 mV pH^–1^ (*I*_DS_ = 200 μA)Response time: < 1 sDrift rate: 0.13 mV pH^–1^ (pH 4)0.38 mV pH^–1^ (pH 7)7.31 mV pH^–1^ (pH 10),Hysteresis width: 4.4 mV (pH 7–4–7–10–7)2.2 mV (pH 7–10–7–4–7)Loop time: ∼13 minInterfering ions: K^+^, Na^+^ (*k* ≈ 4·10^–6^, Equation 4)The *I*_DS_(*U*_G_) curve is shifted positively as the pH value increases (see Figure 3).	[64]
*Coulometric micro-tritrator:* Actuator for the coulometric production: two gold electrodes (on copper support)End-point detection: Ru/RuO_2_ (WE), Ag|AgCl (RE), Au (CE) *Amperometric Biosensor:* 5% Ru/carbon/enzyme (WE) on a silver-conductive layer (CE) on a polyester support *Potentiometric biosensor:* RuO_2_/urease (WE) and RuO_2_/bovine serum albumin (CE) on silver current collector 59.5% RuO_2_, 40% graphite paste, 0.5% urease, screen-printed on a current collector.	Acid-base titration, e.g. 0.01 molar acetic acid: Δ*E* = ∼ 200 mV at 6.8 μA applied current	[65]
	Pesticides monitoring by help of acetylcholine esterase and choline oxidase at 700 mV vs. SCE: Acetylcholine + H_2_O → Acetate + Choline Choline + O_2_ → Betaine aldehyde + H_2_O_2_ The measured current is proportional to choline concentration in phosphate buffer (pH 7).	[66]
	Flow injection system: Dialysate fluid and buffer are continously droped on the sensor by help of a peristalic pump.	[67]
	Detection of silver and copper ions, which inhibit urease, by a change of potential: ∼50 mV mmol^–1^	[68]

## Symbols and Abbreviations

*a*: activity, *a* = γ_c_(*c*/*c*^0^) = γ_m_(*m*/*m*^0^)
*C*: capacitance (F), *C* = d*Q*/d*U*
*c*: molar concentration (mol/L), amount of a substance solved per liter of solution
*c*^0^: standard concentration: *c*^0^ ≡ 1 mol/L
CE: counter electrode
CVD: chemical vapour deposition
*E*: potential of an electrode versus a reference electrode (in V vs. ref)
*E*^0^: standard potential of an electrode or half-reaction (V NHE): at 25 °C, 101325 Pa, 1-active solution
Δ*E*^0^: difference in standard potential of two half-cells (V), electromotive force
*F*: Faraday constant; charge on one mole of electrons: *F* = 96485 C mol^–1^
*I*: electric current (A)
j: imaginary operator, √(–1)
*Q*: electric charge (C = A s)
*M*: molar mass of a compund (kg mol^–1^)
*m*: molality (mol kg^–1^), amount of substance solved per kilogram of solvent: *m* = *c*/(ρ – *Mc*)
*m*^0^: standard molality: *m*^0^ ≡ 1 mol kg^–1^
NHE: standard hydrogen electrode, see Section 2.2
*p*: pressure (1 Pa = 10^–5^ bar)
*p*^0^: standard pressure: 101325 Pa
*R*: electric resistance (Ω)
Ref: Reference (electrode)
RE: reference electrode
RHE: reversible hydrogen electrode: *E*_NHE_ = *E*_RHE_ – 0.05916pH (25 °C)
(s): solid phase
SCE: saturated calomel electrode
SHE: standard hydrogen electrode, see Section 2.2
*T*: absolute temperature (in K)
TRIS: tris(hydroxymethyl) aminomethane
*U*: electric voltage (V), potential difference between two electrodes
Δφ: electric potential difference between two phases (V)
ρ: density of a liquid (1 kg m^–3^ = 1 g/L = 0.001 g cm^–1^)
*v*: scan rate, *v* = d*U*/d*t* (in V s^–1^)
WE: working electrode
*Z*: *ac*, impedance (Ω): *Z* = Re *Z* + j Im *Z*
*z*: charge number of an ion; number of electrons transferred in the half-reaction equation
γ: activity coefficient
